# Components and Insecticidal Activity against the Maize Weevils of *Zanthoxylum schinifolium* Fruits and Leaves

**DOI:** 10.3390/molecules16043077

**Published:** 2011-04-13

**Authors:** Cheng Fang Wang, Kai Yang, Hai Ming Zhang, Jie Cao, Rui Fang, Zhi Long Liu, Shu Shan Du, Yong Yan Wang, Zhi Wei Deng, Ligang Zhou

**Affiliations:** 1 State Key Laboratory of Earth Surface Processes and Resource Ecology, Beijing Normal University, Beijing 100875, China; 2 Department of Entomology, China Agricultural University, Haidian District, Beijing 100094, China; 3 Analytical and Testing Center, Beijing Normal University, Haidian District, Beijing 100875, China; 4 Department of Plant Pathology, China Agricultural University, Haidian District, Beijing 100094, China

**Keywords:** *Zanthoxylum schinifolium*, *Sitophilus zeamais*, fumigant, contact toxicity, essential oil, estragole, linalool, sabinene

## Abstract

In our screening program for new agrochemicals from Chinese medicinal herbs and wild plants, *Zanthoxylum schinifolium* essential oils were found to possess strong insecticidal activity against the maize weevil *Sitophilus zeamais*. The essential oils of *Z. schinifolium* fruits and leaves were extracted via hydrodistillation and investigated by GC and GC-MS. Estragole (69.52%) was the major compound of the essential oil of fresh fruits, followed by linalool (8.63%) and limonene (4.34%) and 94.33% of the total components were monoterpenoids. The main components of the essential oil of fresh leaves were linalool (12.94%), *ar*-tumerone (8.95%), limonene (6.45%) and elixene (5.43%) and only 50.62% were monoterpenoids. However, the essential oil from purchased fruits contained linalool (33.42%), limonene (13.66%) and sabinene (5.72%), followed by estragole (4.67%), nerol (4.56%) and 4-terpineol (4.27%). Estragole, linalool and sabinene were separated and purified by silica gel column chromatography and preparative thin layer chromatography, and further identified by means of physicochemical and spectrometric analysis. The essential oil from the fresh fruits (LD_50_ = 15.93 μg/adult) possessed two times more toxicity to the insects compared with that of fresh leaves (LD_50_ = 35.31 μg/adult). Estragole, linalool and sabinene exhibited contact activity against *S. zeamais* with LD_50_ values of 17.63, 13.90 and 23.98 μg/adult, respectively. The essential oils of *Z . schinifolium* possessed strong fumigant toxicity against *S. zeamais* adults with LC_50_ values of 13.19 mg/L (fresh fruits), 24.04 mg/L (fresh leaves) and 17.63 mg/L (purchased fruits). Estragole, linalool and sabinene also exhibited strong fumigant toxicity against the maize weevils with LC_50_ values of 14.10, 10.46 and 9.12 mg/L, respectively.

## 1. Introduction

Fumigation plays a very important role in insect pest elimination in stored products [1]. Plant essential oils and their components have been shown to possess potential to be developed as new fumigants and they may have the advantage over conventional fumigants in terms of low mammalian toxicity, rapid degradation and local availability [2,3]. Essential oils derived from many plant species have been evaluated so far for fumigant toxicity against stored product insects (for a review, see [4]). During our screening program for new agrochemicals from Chinese medicinal herbs, green prickly ash, *Zanthoxylum schinifolium* Sieb. et Zucc (Family: Rutaceae) was found to possess significant insecticidal activities against the maize weevils (*Sitophilus zeamais* Motsch.).

*Z. schinifolium*, found in China, Japan and Korea, is an aromatic shrub with protective thorns and surprising culinary and pharmacological properties that has been cultivated in the southern provinces of China. In China, this ripe pericarp of the fruits is one of the sources of Perocarpium Zanthoxyli and widely consumed in Asia as a spice [5]. The fruit of this plant has been used in Chinese medicine for epigastric pain accompanied by cold sensation, vomiting, diarrhea and abdominal pain due to intestinal parasitosis, ascariasis and used externally for eczema [6]. The seeds of this species are very flavourful with strong anise, citrus, and pepper notes. Both the pericarp and black seeds are used in cooking. The chemical composition of essential oil derived from *Z. schinifolium* fruits has been widely studied [7,8,9,10]. However, most of the essential oils tested in the previous studies were extracted from the fruits purchased from the local market. In this paper, we report chemical composition of the essential oils derived from the fresh fruits and leaves of *Z. schinifolium*.

Aqueous extracts of *Z. schinifolium* fruitshave been used to control aphids on vegetables [11] and the ethanol extracts of *Z. schinifolium* fruits were found to possess high feeding deterrent activity against two aphids, *Myzus persicae* and *Lipaphis erysimi* [12]. Petroleum ether extracts of *Z. schinifolium* fruits possess strong contact toxicity and repellent activity against the aphids *M. persicae* and the diamond back moth (*Plutella xylostella*) [13,14]. Moreover, two alkaloids were isolated from *Z. schinifolium* fruits and identified that exhibited strong feeding deterrent activity against two stored product insects, *Tribolium castaneum* and *S. zeamais* [15]. In this paper, we report the contact and fumigant toxicity of the crude essential oils and three main components derived from the essential oils against *S. zeamais* adults.

## 2. Results and Discussion

### 2.1. Chemical Composition of the Essential Oils

The GC-MS results of *Z. schinifolium* essential oils are presented in Table 1. A total of 23 components were identified in the essential oil from the fresh fruits (Northeastern China), accounting for 98.56% of the total oil and the essential oil contained 94.33% monoterpenoids and only 4.23% sesquiterpenoids.

**Table 1:** Chemical composition of the essential oils of *Zanthoxylum schinifolium* fruits and leaves.

Compounds	RI *	Fresh Fruits	Fresh Leaves	Purchased Fruits
		Relative content (%)	Relative content (%)	Relative content (%)
α-Pinene	939	0.21	1.31	1.47
β-Thujene	967	0.11	0.21	3.43
Sabinene	977	2.62	3.22	5.72
β-Myrcene	992	2.11	2.04	3.58
(+)-4-Carene	1002	-	-	0.54
Limonene	1029	4.34	6.45	13.66
(*Z*)-Ocimene	1038	1.67	2.07	1.47
γ-Terpinene	1057	0.43	0.06	2.93
Linalool	1097	8.63	12.94	33.42
β-Thujone	1114	-	0.33	0.41
*cis*-ρ-Menth-2-en-1-ol	1126	-	-	0.39
*neo*-Alloocimene	1139	0.04	1.21	0.84
4-Terpineol	1177	2.34	3.56	4.27
γ-Terpinene	1179	0.15	4.06	3.03
α-Terpineol	1189	1.56	2.34	1.36
Estragole	1195	69.52	4.75	4.67
Citronellol	1226	-	1.58	2.71
Nerol	1230	0.52	0.93	4.56
Piperitone	1250	0.08	3.56	1.75
Elixene	1313	0.23	5.43	0.13
β-Geranyl acetate	1381	-	0.07	0.18
β-Elemene	1392	0.62	3.32	0.53
Caryophyllene	1420	0.64	4.49	2.96
γ-Elemene	1437	0.49	1.98	0.13
α-Caryophyllene	1454	0.75	3.28	1.56
Germacrene D	1478	1.15	4.56	2.67
δ-Cadinene	1520	-	0.34	0.27
Spathulenol	1578	0.21	4.59	-
Caryophyllene oxide	1583	0.14	3.66	-
Cubenol	1643	-	2.95	0.05
τ-Muurolo	1662	-	3.22	-
*a r*-Tumerone	1664	-	8.56	-
Total identified		98.56	97.07	98.69
Monoterpenoids		94.33	50.62	90.21
Sesquiterpenoids		4.23	46.45	8.48

* RI, retention index as determined on a HP-5MS column using the homologous series of *n*-hydrocarbons as reference.

Estragole (69.52%) was the major compound followed by linalool (8.63%) and limonene (4.34%). The chemical composition of the essential oil derived from the fresh fruits is quite different from that from the fresh leaves because a total of 30 components were identified in the essential oil from the fresh leaves, accounting for 97.07% of the total oil. The main components, in order of decreasing amounts, were linalool (12.94%), *ar*-tumerone (8.95%), limonene (6.45%) and elixene (5.43%) and 46.45% sesquiterpenoids and 50.62% monoterpenoids were found in the essential oil derived from the leaves (Table 1). However, a total of 28 components were identified in the essential oil from the purchased fruits (Southwestern China), accounting for 98.69% of the total oil. The main components, in order of decreasing amounts, were linalool (33.42%), limonene (13.66%) and sabinene (5.72%) followed by estragole (4.67%), nerol (4.56%) and 4-terpineol (4.27%).

The chemical composition of the essential oils was different from that reported in other studies. For example, the essential oil derived from South Korean plants contained geranyl acetate (29.8%), citronellal (15.8%), and sabinene (15.4%) as major components [16] while Lee *et al*. [9] reported that *β*-phellandrene (22.5%), citronellal (16.5%), and geranyl acetate (11.4%) were identified as the major constituents of the essential oils from South Korean *Z. schinifolium*. However, the essential oil derived from Chinese *Z. schinifolium* fruits was found to have linalool (29%), limonene (14%), and sabinene (13%) as the major components [17] while Jia *et al*. [18] also found that linalool (40.2%), limonene (18.8%), and sabinene (14.5%) were the three main components of essential oil of Chinese *Z. schinifolium* fruits. Great variations were observed in chemical composition of the essential oils of *Z. schinifolium* fruits due to different harvest time and different drying methods [19,20]. The above results showed wide variation in chemical composition and yield of *Z. schinifolium* essential oil, which may be related to the herbal source (climate, soil conditions and geographical location), herbal parts used, chemotype of the plant species, and/or the analytical methods used. The above findings suggest that further studies on plant cultivation and essential oil standardization are needed because the chemical composition of the essential oil of *Z. schinifolium* varies greatly with the plant population.

Sabinene, linalool and estragole were further separated and purified by silica gel column chromatography and preparative thin layer chromatography based on bioassay-guided fractionation and characterized from their ^1^H-, ^13^C-NMR and mass spectra. After comparing the physicochemical and spectrometric data with those reported in the literature [21,22,23,24,25,26], the identities of the three compounds were further confirmed.

### 2.2. Insecticidal Activity

The essential oils of *Z. schinifolium* exhibited strong contact toxicity against maize weevils (Table 2). The essential oils derived from the two kinds of fruits showed the same level of contact toxicity against insects. However, the essential oil from the fresh fruits (LD_50_ = 15.93 μg/adult) possessed two times more toxicity towards the insects compared with that of fresh leaves (LD_50_ = 35.31 μg/adult). The isolated compounds, estragole, linalool and sabinene also exhibited contact toxicity against *S. zeamais* with LD_50_ values of 17.63, 13.90 and 23.98 μg/adult, respectively (Table 2). Another main compound of the essential oils, limonene only exhibited contact toxicity to *S. zeamais* with a LD_50_ value of 29.86 μg/adult [27]. Compared with the famous botanical insecticide, pyrethrum extract (25% pyrethrine I and pyrethrine II), the essential oils were 4–8 times less active against *S. zeamais* adults because pyrethrum extract displayed a LD_50_ value of 4.29 μg/adult [28].

**Table 2:** Contacttoxicity of essential oils of *Zanthoxylum schinifolium* and their main components against *Sitophilus zeamais* adults.

Essential oils	Concentration (%)	LD_50 _(μg/adult)	95% FL	Slope ± SE	Chi square (χ^2^ )
Fresh fruits	1.86-10.00	15.93	13.92-17.96	3.04 ± 0.36	7.84
Fresh leaves	3.72-20.00	35.31	31.19-39.33	3.46 ± 0.37	12.32
Purchased fruits	4.02-10.00	18.55	16.38-20..46	4.67 ± 0.61	13.72
Estragole	1.32-10.00	17.63	15.56-19.97	3.09 ± 0.32	12.04
Linalool	2.00-5.00	13.90	13.05-14.83	5.86 ± 0.55	9.80
Sabinene	2.63-20.00	23.98	21.63-27.15	5.18 ± 0.63	10.08
Limonene*	-	29.86	27.28-30.10	7.38 ± 0.84	8.64
Pyrethrum extract**	-	4.29	3.86-4.72	-	-

* data from Fang *et al.* [27]; ** data from Liu *et al.* [28].

**Table 3:** Fumigant toxicity of essential oils of *Zanthoxylum schinifolium* and their main components against *Sitophilus zeamais* adults.

Essential oils	Concentration (%)	LD_50 _(mg/L air)	95% FL	Slope ± SE	Chi square (χ^2^ )
Fresh fruits	1.32-10.00	13.19	11.63-14.96	3.05 ± 0.31	11.48
Fresh leaves	2.64-20.00	24.04	21.87-26.23	2.59 ± 0.30	8.12
Purchased fruits	2.64-20.00	17.63	15.12-20.21	3.26 ± 0.39	7.13
Estragole	1.32-10.00	14.10	12.45-15.97	3.09 ± 0.32	12.04
Linalool	1.32-10.00	10.46	9.58-11.55	4.20 ± 0.47	10.36
Sabinene	0.9-10.00	9.12	8.17-9.31	2.59 ± 0.32	8.12
Limonene*	-	48.18	45.29-51.04	3.74 ± 0.40	14.88
MeBr**	-	0.67	-	-	-

* data from Fang *et al.* [27]; ** data from Liu and Ho [29].

The essential oils of *Z. schinifolium* also possessed strong fumigant toxicity against *S. zeamais* adults with LC_50_ values of 13.19 mg/L (fresh fruits), 24.04 mg/L (fresh leaves) and 17.63 mg/L (purchased fruits) (Table 3). The isolated compounds estragole, linalool and sabinene also exhibited strong fumigant toxicity against the maize weevils with LC_50_ values of 14.10, 10.46 and 9.12 mg/L, respectively (Table 3). Limonene was reported to possess fumigant toxicity against *S. zeamais* adults with a LC_50_ value of 48.18 mg/L [28]. The commercial grain fumigant, methyl bromide (MeBr) was reported to have fumigant activity against *S. zeamais* adults with a LC_50_ value of 0.67 mg/L [29], thus the essential oils were 20–36 times less toxic to *S. zeamais* adults compared with MeBr. However, compared with the other essential oils in the literature, the essential oils of *Z. schinifolium* exhibited the same level of fumigant toxicity against the maize weevils, e.g. essential oils of *M. exotica* (LC_50_ = 8.29 mg/L) [30], *Artemisia lavandulaefolia* (LC_50_ = 11.2 mg/L) [28], *A. vestita* (LC_50_ = 13.42 mg/L) [31], *Illicium simonsii* (LC_50_ = 14.95 mg/L) [32] and *A. sieversiana* (LC_50_ = 15.0 mg/L) [28].

In the previous reports, linalool was also found to have fumigant toxicity against the triatomine bug (*Rhodnius prolixus*) [33] and houseflies with a 24 h LC_50_ value of 13.6 mg/L air [34]. Moreover, linalool possessed both contact and fumigant toxicity against human head louse (*Pediculus humanus capitis*) [35] and showed a high acaricidal activity by vapor action against mobile stages of *Tyrophagus putrescentiae* [36]. Linalool was found to be a competitive inhibitor of acetyl-cholinesterase (AchE) [37]. Estragole was also demonstrated to exhibit contact and fumigant toxicity against several insects and mites, e.g. the houseflies [34], fruit flies (*Ceratitis capitata*, *Bactrocera dorsalis*, and *Bactrocera cucurbitae*) [38], house dust mites [39,40], stored product insects [41] and grasshoppers [26]. Considering the currently used fumigants are synthetic insecticides, fumigant activity of the crude essential oils and the three isolated compounds are quite promising and they show potential for development as possible natural fumigants for the control of stored product insects. For the practical use of *Z. schinifolium* essential oils and their constituents as novel fumigants or insecticides to proceed, further research is needed to establish their human safety. However, in traditional Chinese medicine, prickly ash is used in many chronic problems such a rheumatism and skin diseases; chilblains, cramp in the leg, varicose veins and varicose ulcers. It is also used for low blood pressure, fever, and inflammation. Externally it may be used as a stimulation liniment for rheumatism and fibrositis [6]. It seems that this medicinal herb is quite safe for human consumption because it has been used as a spice for hundreds of years. However, no experimental data about the safety of this herb is available so far. Additionally, their fumigant and insecticide modes of action need to be established and formulations for improving insecticidal potency and stability, thereby reducing costs, need to be developed.

## 3. Experimental

### 3.1. General

^1^H- and ^1^^3^C-NMR spectra were recorded on Bruker Avance DRX 500 instruments using CDCl_3_ as solvent with TMS as internal standard. EIMS were determined on a ThermoQuest Trace 2000 mass spectrometer at 70 eV (probe). Silica gel (160–200 mesh) and pre-coated GF_254_ plates were purchased from Qingdao Marine Chemical Plant (Shandong Province, China). Fluon was purchased from ICI America Inc (USA). C_8_-C_24_ *n*-alkanes were purchased from Sigma-Aldrich (USA). All other unlabelled chemicals and reagents were of analytical grade.

### 3.2. Plant Material

Dried fruits of *Z. schinifolium* (2 kg, harvested from Jinyang, Sichuan Province, China) were purchased from Anguo Chinese Medicinal Herbs Market (Anguo 071200, Hebei Province, China). Fresh fruits (10 kg) and leaves (10 kg) of *Z. schinifolium* were collected at September 2010 from Anshan 114000, Liaoning Province, China. The voucher specimens (BNU-CMH-Dushuahan-2009-09-23-006, BNU-CMH-Dushushan-2010-08-18-003) were deposited at the Herbarium (BNU) of College of Life Sciences, Beijing Normal University. The fresh fruits and leaves were air-dried for one week. The fruits and leaves were ground to powder using a grinding mill (Retsch Muhle, Germany).

### 3.3. Insects

The maize weevils (S. zeamais) were obtained from laboratory cultures maintained in the dark in incubators at 29–30 °C and 70–80% r.h. The maize weevils were reared on whole wheat at 12–13% moisture content. Unsexed adult weevils used in all the experiments were about 2 weeks old.

### 3.4. Essential Oil Distillation

The ground powder of *Z. schinifolium* fruits and leaves was subjected to hydrodistillation for 6 h using a modified Clevenger-type apparatus. Anhydrous sodium sulphate was used to remove water after extraction. Essential oils were stored in airtight containers in a refrigerator at 4 °C. The oil yields of fresh fruits and purchased fruits were 6.92% and 8.14% v/w, respectively. The oil yields of fresh leaves were only 0.85% v/w.

### 3.5. Purification and Characterization of Sabinene, Linalool and Estragole

The crude essential oil of the purchased fruits was chromatographed on a silica gel column by gradient elution with *n*-hexane first, then with *n*-hexane-ethyl acetate, and last with acetone to obtain 20 fractions. Of these, fraction 5 and 7 were further separated by PTLC with petroleum ether-acetone (50:1, v/v) to afford two pure compounds, sabinene (**1**) and linalool (**2**), respectively. The crude essential oil of the fresh fruits was also chromatographed on a silica gel column by gradient elution to obtain estragole (**3**).

**Figure 1:**
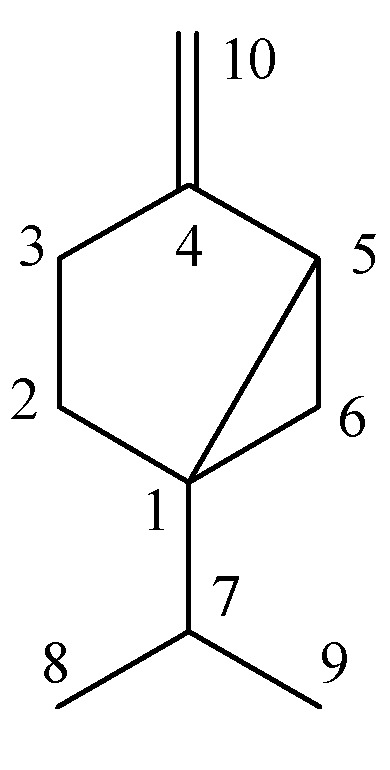
Sabinene.

*Sabinene* (**1**, Figure 1). Colorless liquid, ^1^H-NMR (CDCl_3_) δ (ppm): 4.83 (1H, s, H-10), 4.65 (1H, s, H-10), 2.01–2.21 (2H, m, 3-CH_2_), 1.72–1.78 (2H, m, 2-CH_2_), 1.62(1H, m, H-5), 1.52(1H, m, H-7), 0.98 (3H, d, *J* = 6.81 Hz, 8-CH_3_), 0.92 (3H, d, *J* = 6.81 Hz, 9-CH_3_), 0.69 (1H, d, *J* = 5.2 Hz, 6-CH_2_), 0.68 (1H, d, *J* = 5.2 Hz, 6-CH_2_). ^13^C-NMR (CDCl_3_) δ (ppm): 154.38 (C-4), 101.52 (C-10), 37.63 (C-1), 32.58 (C-7), 30.12 (C-5), 28.96 (C-3), 27.46 (C-2) 19.79 (C-8), 19.70 (C-9), 16.03 (C-6). Its identity was confirmed by the EI-MS with the mass spectral fragmentation pattern [*m/z* (% abundance): 136 (M^+^, 16), 121 (25), 93 (100), 91 (17), 79 (22), 77 (30), 69 (16), 41 (18), 39 (10)]. The data matched with previous reports [21,23,24].

**Figure 2:**
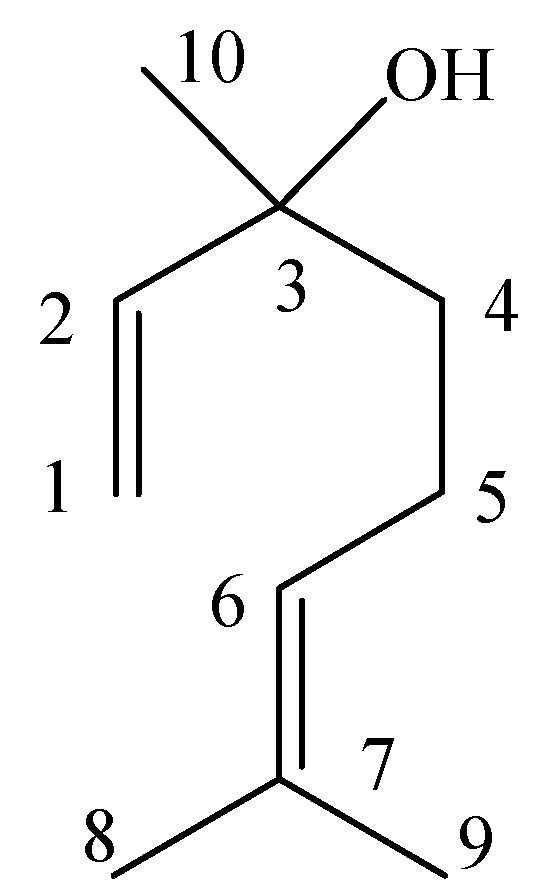
Linalool.

*Linalool* (**2**, Figure 2). Colorless oil, ^1^H-NMR (_,_CDCl_3_) δ (ppm): 5.90 (1H, dd, *J* = 17.3, 10.8 Hz, H-2), 5.21 (1H, d, *J* = 17.3 Hz, H-1,trans), 5.12(1H, t, H-6), 5.05 (1H, d, *J* = 10.7 Hz, H-1,cis), 2.01 (2H, m, 5-CH_2_), 1.83 (1H, br, 3-OH, which was removed in the deuterium exchange spectrum after adding a drop of deuterated water), 1.68 (3H, s, 9-CH_3_), 1.60 (3H, s, 8-CH_3_), 1.55 (2H, m, 4-CH_2_), 1.27 (3H, s, 10-CH_3_). ^13^C-NMR (CDCl_3_) δ (ppm): 145.03 (C-2), 131.81 (C-7), 124.33 (C-6), 111.63 (C-1), 73.40 (C-3), 42.05 (C-4), 27.79 (C-10), 25.66 (C-8), 22.77 (C-5), 17.65 (C-9). EI-MS *m/z* (%): 154 (M^+^, 5), 136 (15), 121 (25), 109 (11), 93 (80), 83 (18), 80 (30), 71 (100), 69 (50), 55 (45), 43 (39), 41(62), 27 (15). The data matched with previous reports [21,22].

**Figure 3:**
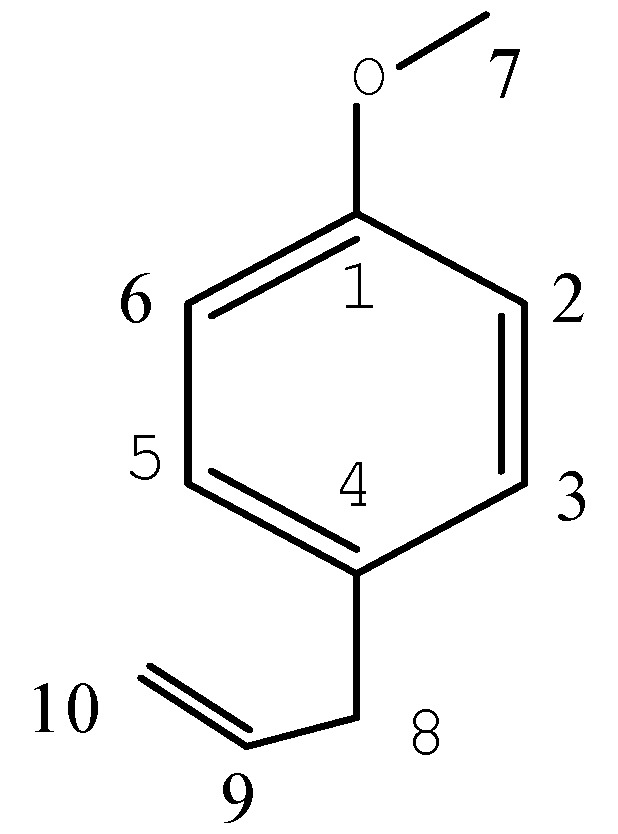
Estragole.

*Estragole* (**3**, Figure 3). Colorless oil, ^1^H-NMR (CDCl_3_) δ (ppm): δ 7.27 (1H, d, H-3, 5), 7.01 (1H, d, H-2, 6), 6.14 (1H, m, H-9), 5.26 (2H, d, H-10), 3.92 (3H, s, 7- CH_3_), 3.50 (2H, d, H-8). ^13^C-NMR (CDCl_3_) δ (ppm): 158.22 (C-9), 138.08 (C-1), 132.16 (C-10), 129.61 (C-4), 115.52 (C-3, C-5), 113.99 (C-2, C-6), 55.18(C-7), 39.52 (C-8). EI-MS *m/z* (%): 149.0(13), 148 (100), 147 (46), 133 (23), 121 (41), 117 (32), 115 (16), 105 (23), 103 (11), 91 (20), 79 (13), 79 (13), 78 (15), 77 (23), 51 (12), 9 (11). The data matched with previous reports [21,25,26].

### 3.6. Gas Chromatography and Mass Spectrometry

Gas chromatographic analysis was performed on an Agilent 6890N instrument equipped with a flame ionization detector and an HP-5MS (30 m × 0.25 mm × 0.25 μm) capillary column, while the essential oil components were identified on an Agilent Technologies 5973N mass spectrometer. The GC settings were as follows: the initial oven temperature was held at 60 °C for 1 min and ramped at 10 °C min^−1^ to 180 °C for 1 min, and then ramped at 20 °C min^−1^ to 280 °C for 15 min. The injector temperature was maintained at 270 °C. The samples (1 μL) were injected neat, with a split ratio of 1:10. The carrier gas was helium at flow rate of 1.0 mL min^−^^1^. Spectra were scanned from 20 to 550 *m/z* at 2 scans s^−1^. Most constituents were identified by gas chromatography by comparison of their retention indices with those of the literature or with those of authentic compounds available in our laboratories. The retention indices were determined in relation to a homologous series of *n*-alkanes (C_8_–C_24_) under the same operating conditions. Further identification was made by comparison of their mass spectra with those stored in NIST 05 and Wiley 275 libraries or with mass spectra from literature [42]. Component relative percentages were calculated based on GC peak areas without using correction factors.

### 3.7. Fumigant Toxicity

The fumigant activity of the essential oil and the pure compounds against *S. zeamais* adults was tested as described by Liu and Ho [29]. Range-finding studies were run to determine the appropriate testing concentrations. A serial dilution of the essential oil/compound (six concentrations) was prepared in *n*-hexane. A Whatman filter paper (diameter 2.0 cm) were each impregnated with 10 μL dilution, and then placed on the underside of the screw cap of a glass vial (diameter 2.5 cm, height 5.5 cm, volume 25 mL). The solvent was allowed to evaporate for 15 s before the cap was placed tightly on the glass vial, each of which contained 10 insects inside to form a sealed chamber. Fluon (ICI America Inc) was used inside glass vial to prevent insects from contacting the treated filter paper. Preliminary experiments demonstrated that 15 s was sufficient for the evaporation of solvents. *n*-Hexane was used as a control. Five replicates were carried out for all treatments and controls, and they were incubated for 24 h. The insects were then transferred to clean vials with some culture media and returned to the incubator and observed daily for determination of end-point mortality, which was reached after one week. The experiments were repeated in three times. The LC_50_ values were calculated by using Probit analysis [43].

### 3.8. Contact Toxicity

The contact toxicity of the essential oil/pure compounds against *S. zeamais* adults was measured as described by Liu and Ho [29]. Range-finding studies were run to determine the appropriate testing concentrations. A serial dilution of the essential oil/compound (six concentrations) was prepared in *n*-hexane. Aliquots of 0.5 μL of the dilutions were applied topically to the dorsal thorax of the insects. Controls were determined using *n*-hexane. Five replicates were carried out for all treatments and controls. Both treated and control insects were then transferred to glass vials (10 insects/vial) with culture media and kept in incubators. Mortality of insects was observed daily until end-point mortality was reached one week after treatment. The experiments were repeated in three times. The LD_50_ values were calculated by using Probit analysis [43].

## 4. Conclusions

Identified through mass screening, essential oils of *Z. schinifolium* fruits and leaves and their major components were examined for insecticidal activity against the maize weevil *S. zeamais*. The essential oils possessed strong fumigant toxicity against *S. zeamais* adults and they were 20 times less toxic compared to the commercial fumigant MeBr. The three isolated compounds also exhibited strong fumigant toxicity against *S. zeamais* adults. The essential oils and the three compounds also showed contact toxicity against *S. zeamais*. These findings, considered together, suggest that the essential oils and the three compounds show potential for development as natural fumigants for stored products.
